# Metastatic non-muscle invasive bladder cancer with cervical lymph node metastasis

**DOI:** 10.1590/S1677-5538.IBJU.2018.0863

**Published:** 2019-12-17

**Authors:** Pablo Garrido-Abad, Luis García Martín, Karen Villar Zarra, Ariel Díaz Menéndez, Manuel Fernández Arjona

**Affiliations:** 1 Department of Urology, Hospital Universitario del Henares, Coslada, Universidad Francisco de Vitoria, Madrid, Spain;; 2 Department of Pathology, Hospital Universitario del Henares, Coslada, Universidad Francisco de Vitoria, Madrid, Spain

**Keywords:** Urinary Bladder Neoplasms, Neoplasm Metastasis, Lower Urinary Tract Symptoms

## Abstract

Bladder cancer is a common cancer that may present as superficial, invasive, or metastatic disease. Non-muscle-invasive bladder cancer (NMIBC) represents the majority of bladder cancer diagnoses, but represents a spectrum of disease with a variable clinical course, notably for significant risk of recurrence and potential for progression. NMIBC metastasis to distant organs without local invasion or regional metastasis is a very rare occurrence, so there are limited case reports about early metastasis in the literature.

## CASE REPORT

**Figure 1:**
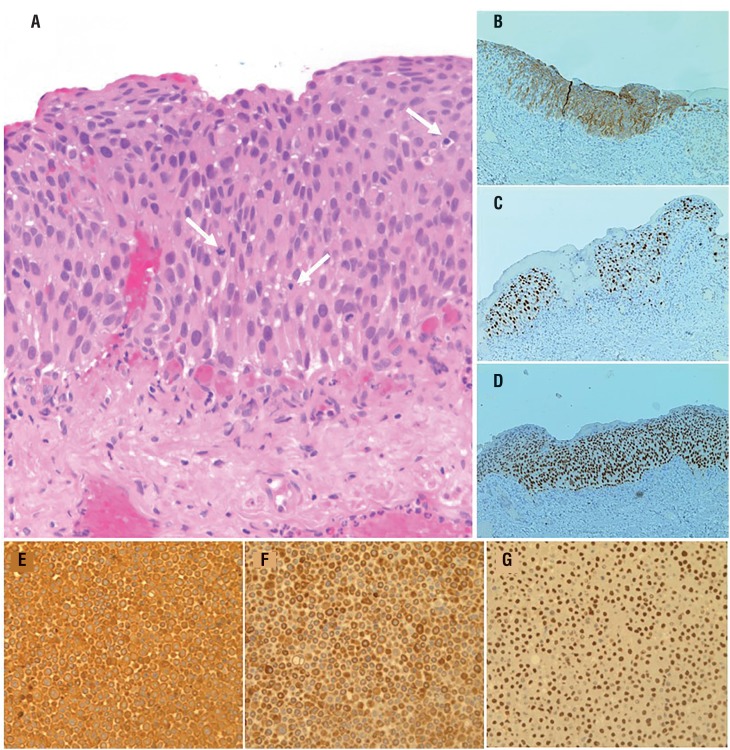
Histopathology of the bladder after TURBT: (A) H-E stain (20x) with high-grade urothelial CIS. (B) Strong full thickness expression of cytokeratin 20. (C) High proliferative activity measured by Ki67 index. (D) Strong and diffuse overexpression of p53 protein (positivity was seen throughout urothelial thickness). Immunochemistry after fine needle aspiration cytology of the LNM: intense positivity for CK7 (E) CK20 (F) and GATA-3 expression (G). (Magnification of 400X).

**Figure 2:**
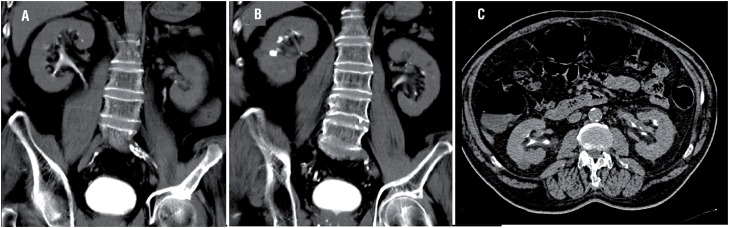
(A, B) Coronal and (C) axial scans of computed tomography (CT) urography without signs of UUT disease.

A 72-year-old man with diabetes was admitted in January 2017 with complaints of lower urinary tract symptoms and microscopic hematuria. Review of the medical history showed that in 2012 he had undergone transurethral resection of a low-grade pTa bladder tumor (TURBT), without signs of recurrence until February 2017 when atypical urothelial cells were described by urine cytology and a small superficial bladder neoplasm was found on cystoscopy. Hence, the patient was treated again with TURBT. At this time the pathological diagnosis was secondary carcinoma in situ (CIS) (Figures 1A-D), that was confirmed by an additional pathologist review, so he was treated with 81mg of intravesical bacillus Calmette-Guerin (BCG) once weekly for 6 weeks+3 weekly instillations every 3 months. Following the protocol of high-risk bladder cancer, upper urinary tract (UUT) was evaluated with computed tomography (CT) urography without signs of UUT disease (Figure 2). Thus, a second TUR (Re-TUR) with random bladder biopsies was performed 45 days later, with non-tumoral bladder tissue in histopathology.

**Figure 3:**
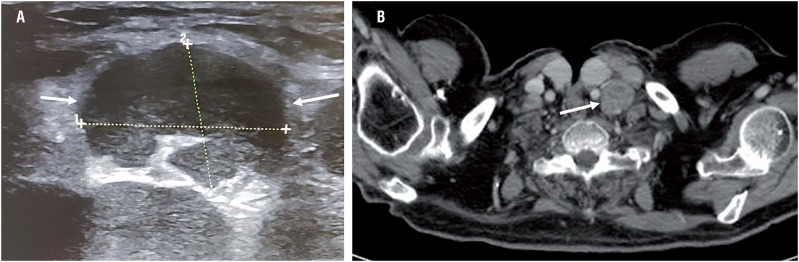
(A) Ultrasound of the left supraclavicular region and (B) contrast-enhanced computed tomography scan showing enlarged left supraclavicular LNs (arrows).

Postoperatively, the patient was followed with cystoscopy and urinary cytology every three months and remained free of recurrence for 8 months, when he complained about a left cervical mass. A cervical US (Figure 3A) and full-body CT scan (Figure 3B) revealed several left supraclavicular lymph node (LN) enlargement without evidence of local or regional (pelvic) recurrence. Fine-needle aspiration cytology (FNAC) of LNs was performed under US guidance. The immunochemical staining showed diffuse co-expression of cytokeratin 7 (Figure 1E), cytokeratin 20 (Figure 1F) and GATA-3 (Figure 1G), with negative expression of TTF1 and napsin A, that definitely identified metastatic UC. He received 6 cycles of combination chemotherapy (gemcitabine-cisplatin) every 4 weeks, with a progressive clinical worsening, complicated by a fatal acute respiratory failure 5 months later.

## DISCUSSION

Bladder cancer (BC) is the seventh most commonly diagnosed cancer in the male population worldwide (1). Approximately 75% of patients with BC present with a disease confined to the mucosa (Ta, CIS), or submucosa (T1), being the urothelial carcinoma (UC), the most frequent histological subtype (2). NMIBC prognosis is based on the pathologic findings regarding tumor grade, multiplicity, size, concomitant CIS, depth of invasion, and early recurrence, but are not adequately accurate in predicting the individual clinical behavior (3). TURBT is the initial critical step in the management and staging of disease. An inadequate resection of the initial tumor (inadequate sampling of muscularis propria or missing tumors such as CIS) can lead to an improper staging and early recurrences (4). Half of all NMIBC patients will experience tumor recurrence within 5 years, and 20-30% will progress to secondary muscle-invasive bladder cancer (5). Ultimately, as many as 10-15% of patients presenting with NMIBC will die of bladder cancer (6), with progression-free 5-year survival rate of 77% and an overall survival rate of 63% after 5 years (7).

CIS of the bladder is a flat, high-grade, non-invasive UC comprising about 10% of all cases of BC. The management of CIS continues to present with many challenges, although intravesical BCG has been accepted as first-line therapy, with overall response rate of 86.6% and a 5-year progression-free survival rate of 78.5% (8), this can be complicated by recurrence and progression. Recent study showed that 12% of CIS had a regional LN invasion after radical cystectomy (9), so EAU guidelines also recommend radical cystectomy (10), mainly in patients with BCG refractory high risk NMIBC, rapid relapsing or refractory disease (<6 months), T1 disease on repeat transurethral resection, high-volume multifocal high-grade disease, presence of lymphovascular invasion with T1 disease, unfavorable or mixed histology, and poor patient compliance, because they are at significant risk of progression and metastasis (11, 12). The current role of early cystectomy in CIS is controversial, some altered gene expression as high mRNA expression of ERBB2 is suggested to be useful to identify a high-risk group for progression who will benefit from an early cystectomy (13), although the potential for overtreatment must also be considered.

This is a case of initially diagnosed NMIBC that developed early, multiple cervical metastases. It is very unusual for a patient with NMIBC to develop head and neck metastasis without regional LNM or local invasion. It is difficult to establish the exact lymphatic pathways to the neck nodes from primary tumors arising below the clavicles, although there is a predilection for such metastases to be located to the left neck, while involvement of right-sided neck nodes may be associated with more extensive mediastinal involvement. Rarely, cancers originating from sites other than head and neck can metastasize to the cervical LN chain. However, genitourinary tract tumors, make up a significant proportion of these cancers and should be considered in the differential diagnosis of metastatic lesions of the head and neck (14). CIS metastases to distant organs without local invasion or regional metastases are a very rare occurrence, so there are limited case reports in the literature (3, 15, 16).

Because of the low cost and high accuracy, the combination of US and FNAC represents a valuable and reliable method of choice for diagnosis in most cases of enlarged LNs from an unknown primary (17). Immunohistochemical as well as molecular studies can render a more accurate tissue diagnosis and can be carried out on cytology samples, being GATA-3 the most helpful immune stain for UC (17). Attempts to improve LN staging have shifted imaging towards increased use of MRI, which has a better soft tissue resolution (being able to detect LNM in normal sized LNs) than conventional CT (18). Routine use of F--fluorodeoxyglucose (18F-FDG) positron emission tomography (PET)/CT is still not recommended in bladder cancer staging or in LNM detection (18), however, its use is becoming more common due to recent reports suggesting that PET/CT and SU-Vmax interpretation is an appropriate tool in the evaluation of LN and can be used prior to surgery to make a safe identification of positive LNs (PET provides information on the potential malignancy of a LN through glucose metabolism by 18F-FDG uptake) (19).

Metastatic BC remains an aggressive disease associated with limited treatment options and reduced survival. As many as 20% of patients will ultimately die of metastatic disease, and a significant proportion will develop both intravesical and extravesical recurrences (4), being LNs, bones, and lung the three most common sites of metastasis.

Standard treatment for metastatic cervical LN BC is not well established due to few cases reported, however, palliative radiation therapy and chemotherapy has been offered (19). There is no current reported evidence to support therapeutic neck dissection for the management of BC metastases to the neck (17,20).

Some biomarkers, like p53 and Ki-67, are well studied and found to be correlated with progression in BC (21). Meanwhile, diabetic patients with NMIBC present more early distant metastases than expected, although they are non-invasive (22). For all this, high-risk patients with marked alterations in combined biomarker expression may benefit more from immediate radical cystectomy.
